# Supplementary material for: Determination of the Ecotoxicity of Herbicides Roundup® Classic Pro and Garlon New in Aquatic and Terrestrial Environments
Source: Plants (Basel). 2020 Sep 14;9(9):1203. doi: 10.3390/plants9091203 (PMC7569783; doi:10.3390/plants9091203)
Supplement: Supplementary file 1 [file plants-09-01203-s001.pdf]

Supplementary materials

For the manuscript “Determination of the Ecotoxicity of Herbicides Roundup and Garlon in Aquatic and Terrestrial Environments”.

1. DHA: glyphosate concentration

Table S1: DHA – glyphosate concentration

Concentration of added solution (%)	100	75	50	25	12,5	6,2
Concentration of glyphosate in soil (μg·g^{-1})	135.0	101.3	67.5	33.8	16.9	8.4

2. Statistical analysis

Table S2a. One-way analysis of variance for determination whether group mean differences exist in the values of dehydrogenase activity between the particular herbicides (p = 0.05) and ANOVA post-hoc Tukey-Kramer multiple comparison test (F_{crit} = 0.077). The numbers represent the F-values of significancy obtained from the test.

**ANOVA**			
Between groups	0.011		
**Tukey-Kramer**			
	Roundup® Classic Pro	AMPA	Glyphosate
Roundup® Classic Pro	x	0.038	0.102*
AMPA	x	x	0.065

Means followed by the “*” sign differ by the Tukey - Kramer test ($F>F_{crit}$).

Table S2b. Kruskal-Wallis test of variance for determination whether the group population medians are equal (or the group populations have equal dominance). The numbers represent the H-values of significancy obtained from the test.

	**H**	**H_{crit}**
Duckweed	1.977	5.853
Chlorophyll	0.739	5.902
D. subspicatus	11.363*	5.902

Means followed by the "*" sign differ by the Kruskal-Wallis test ($H>H_{crit}$).

Table 2c. Kruskal-Wallis post-hoc Dunn's test for *D. subspicatus* (p = 0.05; Bonferroni correction, p_{corr} = 0.0167). The numbers represent the F-values of significancy obtained from the test.

	Roundup® Classic Pro	AMPA	Glyphosate
Roundup® Classic Pro	x	0.813	0.005*
AMPA	x	x	0.002*

Means followed by the "*" sign differ by the Dunn's test ($F<p_{corr}$).

3. pH values

Table S3: pH values

**AMPA – pH value**										
Concentration of AMPA (mg·L^{-1})	2852	2139	1426	713	356	178	86	43	21	11
pH of AMPA solution	3.67	3.75	3.80	3.90	4.08	4.16	4.30	4.43	4.64	4.81
Algae inhibition test, pH after 72 h	4.37	4.56	4.63	5.40	5.67	6.50	6.93	7.39	7.63	7.71
Duckweed inhibition test, pH after 7 d	4.49	4.56	4.75	4.82	5.07	5.62	5.75	5.85	5.91	5.85

4. Inhibition values corresponding to all of the tested concentrations

Table S4 a: Algae toxicity test

**Algae (*D. subspicatus*) toxicity test**									
	Roundup® Classic Pro			Garlon New			AMPA		
%	c (µg·L^{-1})	log c	I (%)	c (µg·L^{-1})	log c	I (%)	c (mg·L^{-1})	log c	I (%)
100.00	18,381.0	4.2	85.1	15,000.0	4.1	91.9	2,851.6	6.4	89.6
75.00	13,785.7	4.1	84.3	11,250.0	4.0	90.4	2,138.7	6.3	88.4
50.00	9,190.5	3.9	83.2	7,500.0	3.8	90.4	1,425.8	6.1	88.6
25.00	4,595.2	3.6	82.1	3,750.0	3.5	89.8	712.9	5.8	87.0
12.50	2,297.6	3.3	82.3	1,875.0	3.2	90.9	356.4	5.5	89.6
6.25	1,148.8	3.0	81.6	937.5	2.9	87.8	178.2	5.2	81.7
3.12	574.4	2.7	77.0	468.7	2.6	88.4	128.3	5.1	67.2
1.56	287.2	2.4	75.4	234.3	2.3	88.9	85.5	4.9	6.2
0.78	143.6	2.1	63.8	117.1	2.0	90.0	64.1	4.8	4.7
0.39	71.8	1.8	50.9	58.5	1.7	88.6	42.7	4.6	0.9
0.19	35.9	1.5	16.1	29.2	1.4	83.4	21.3	4.3	-5.7
0.09	17.9	1.2	7.1	14.6	1.1	71.4	10.6	4.0	-3.1
0.04	8.9	0.9	2.0	7.3	0.8	12.1	5.3	3.7	-3.7
0.02	4.4	0.6	-0.1	3.6	0.5	9.5	2.6	3.4	-4.5
0.01	2.2	0.3	-5.8	1.8	0.2	10.3	1.3	3.1	-5.6

Table S4 b: Duckweed toxicity test

**Duckweed (*L. minor*) toxicity test**									
	Roundup® Classic Pro			Garlon New			AMPA		
%	c (µg·L^{-1})	log c	I (%)	c (µg·L^{-1})	log c	I (%)	c (mg·L^{-1})	log c	I (%)
100	18,381.0	4.2	100.0	15,000.0	4.1	100.0	2,851.6	6.4	100.0
75	13,785.7	4.1	100.0	11,250.0	4.0	100.0	2,138.7	6.3	100.0
50	9,190.5	3.9	100.0	7,500.0	3.8	100.0	1,425.8	6.1	100.0
25	4,595.2	3.6	100.0	3,750.0	3.5	100.0	712.9	5.8	100.0
12.5	2,297.6	3.3	100.0	1,875.0	3.2	100.0	356.4	5.5	99.2
6.2	1,148.8	3.0	100.0	937.5	2.9	100.0	178.2	5.2	86.1
3.1	574.4	2.7	94.0	468.7	2.6	100.0	85.5	4.9	85.2
1.5	287.2	2.4	84.9	234.3	2.3	100.0	42.7	4.6	84.7
0.7	143.6	2.1	70.5	117.1	2.0	73.0	21.3	4.3	70.7
0.3	71.8	1.8	55.3	58.5	1.7	42.5	10.6	4.0	36.8
